# Utilization of psychotropic drugs in Serbia from 2006 to 2021: Patterns before and during the COVID-19 pandemic

**DOI:** 10.1371/journal.pone.0330749

**Published:** 2025-09-24

**Authors:** Jelena Filimonovic, Milena Stevanovic, Tatjana Gazibara, Vladan Saponjic, Jelena Dotlic, Perisa Simonovic, Ivana Vukajlovic, Mirjana Stojanovic Tasic, Bojan Joksimovic, Maja Stosic, Miljan Adamovic, Marina Jelic, Jelena Perovic, Danijela Nastic Simovic, Ana Adamovic, Anastasija Karapandzic, Dajana Nogo Zivanovic, Zorica Stanojevic Ristic, Marija Milic

**Affiliations:** 1 Department of Epidemiology, Faculty of Medicine, University of Pristina Temporarily Settled in Kosovska Mitrovica, Kosovska Mitrovica, Serbia; 2 Public Health Institute of Kosovska Mitrovica, Kosovska Mitrovica, Serbia; 3 Clinic of Psychiatry, University Clinical Centre of Serbia, Belgrade, Serbia; 4 Faculty of Medicine, Institute of Epidemiology, University of Belgrade, Belgrade, Serbia; 5 Institute of Public Health of Serbia “Dr Milan Jovanovic Batut”, Belgrade, Serbia; 6 Faculty of Medicine, University of Belgrade, Belgrade, Serbia; 7 Clinic for Obstetrics and Gynecology, Clinical Center of Serbia, Belgrade, Serbia; 8 Medicines and Medical Devices Agency of Serbia (ALIMS), Belgrade, Serbia; 9 Department of Psychiatry, Faculty of Medicine, University of Pristina Temporarily Settled in Kosovska Mitrovica, Kosovska Mitrovica, Serbia; 10 Faculty of Medicine Foca, University of East Sarajevo, Sarajevo, Republic of Srpska (Bosnia and Herzegovina); 11 Faculty of Business Economy, Eductons University, Sremska Kamenica, Serbia; 12 Faculty of Medical Sciences, University of Kragujevac, Kragujevac, Serbia; 13 Department of neurology, Clinical Hospital Center of Kosovska Mitrovica, Kosovska Mitrovica, Serbia; 14 Institution for Accommodation of Adults in Male Pčelica Kragujevac, Kragujevac, Serbia; 15 Student at Faculty of Medicine, University of Pristina Temporarily Settled in Kosovska Mitrovica, Kosovska Mitrovica, Serbia; 16 Department of Pharmacology, Faculty of Medicine, University of Pristina Temporarily Settled in Kosovska Mitrovica, Kosovska Mitrovica, Serbia; University of Thessaly Faculty of Medicine: Panepistemio Thessalias Tmema Iatrikes, GREECE

## Abstract

**Background:**

The increasing global prevalence of mental disorders as well as a persistent stigma make mental disorders a public health priority. The aim of this study was to provide a comprehensive overview of psychotropic drugs utilization from 2006 to 2021 in the Republic of Serbia, examining both pre pandemic and pandemic-related changes.

**Methods:**

To conduct this descriptive study, publicly available data on psychotropic drugs were retrieved from the official website of the Agency for Medicines and Medical Devices of Serbia (ALIMS). The linear and joinpoint regression were used in data analysis.

**Results:**

A total of 54 psychotropic drugs use was analyzed from 2006 to 2021. There was an increase in the consumption of antidepressants, atypical antipsychotics, anxiolytics, sedatives, hypnotics, anti-dementia drugs and gabapentinoid-based drugs. The increase in the consumption of the psychotropic drugs was linear, with no differences between the pre-COVID-19 period and the COVID-19 pandemic. Contrary, a significant decrease in use was observed for some antidepressants (maprotiline, moclobemide, mianserin), antipsychotics (chlorpromazine, fluphenazine), psychostimulants and nootropic drugs (piracetam), anxiolytics (diazepam, prazepam), sedatives and hypnotics (midazolam).

**Conclusion:**

The COVID-19 pandemic did not contribute to change in consumption of psychotropic drugs in Serbia. Still, the use of antidepressants, atypical antipsychotics, anxiolytics, sedatives, hypnotics, anti-dementia drugs and gabapentinoids increased from 2006 to 2021.

## Introduction

The increasing global prevalence of mental disorders and persistent stigmatization of people affected make mental disorders a public health priority [1–3]. Worldwide, depression and anxiety are among the top ten causes of disability in people aged 10–49 years [3]. Unfortunately, diagnosed mental disorders are only the tip of the iceberg, as many psychological disturbances remain unrecognized. The trajectory of treated mental health disorders can be identified through the use of dispensed psychotropic medications.

A recent study examining psychotropic drug utilization over the past 12 years in 65 countries suggested an average annual increase in psychotropic drug use of 4% [3]. The highest annual increase was recorded for antidepressants (3.50%) and antipsychotics (2.49%), while a slight decrease was registered for tranquilizers (−0.99%) and sedatives-hypnotic drugs (−0.91%) [3]. There are remarkable differences in utilization of psychotropic drugs between countries. For example, consumption of psychotropic drugs in lower and upper-middle-income countries is lower than in high-income countries [3]. This can be attributed to the fact that there is a large gap in recognition, early detection and treatment of mental disorders worldwide, as well as that between 76% and 85% of people with severe mental disorders in low- and middle-income countries do not receive treatment, as opposed to high income countries (35% and 50%) [4].

The COVID-19 pandemic has put a tremendous mental health burden worldwide [5]. This may be the reason for a remarkable increase in mental health disturbances since the beginning of the pandemic [6]. Specifically, in Western European countries and the United States (US), the prevalence of depression and anxiety doubled during the pandemic period [6,7]. A recent study suggested a 27.6% increase in unipolar or major depressive disorder and a 25.6% increase in anxiety disorders during the first year of the COVID-19 pandemic [8]. In addition to depression and anxiety, an increase in the frequency of insomnia was also evident, and was especially prominent in the first months of the pandemic [9].

Over the last 20 years, people in Serbia have been exposed to a large number of stressors, such as the unrest in the Kosovo province, economic transition as well as the COVID-19 pandemic. All these factors may have had a negative impact on mental health of the population [10]. It is estimated that one in six adults in Serbia has a high risk of exposure to stress, while one in 20 has a moderate risk of suffering from a mental disorder [11]. Therefore, high rates of psychotropic drugs utilization, especially that of tranquilizers (94.50 defined daily doses/1000/day), were observed in the latest global report which included data from Serbia [3]. Similar results were obtained in a study from 2014 to 2018 conducted in several Balkan countries, suggesting that differences in the economic development and socio-economic factors contribute to a higher benzodiazepines use [12].

Utilization of psychotropic drugs has been extensively discussed among health care professionals and in the general public, because there is evidence that these medications are being used excessively [13]. Systematic monitoring of psychotropic drug utilization trends is essential to better understand changes in mental health care needs over time. Therefore, the aim of this study was to provide a comprehensive overview of psychotropic drugs utilization from 2006 to 2021 in the Republic of Serbia, examining both pre pandemic and pandemic-related changes.

## Method

### Study design and data collection

To conduct this descriptive study, publicly available data were retrieved from the official website of the Medicines and Medical Devices Agency of Serbia (ALIMS). In accordance with the Law on Medicines and Medical Devices of the Republic of Serbia (RS), the ALIMS is responsible for collecting, processing and publishing data on the use of medicines every year at a national level. To describe the pharmaco-economic and pharmaco-epidemiological indicators, data are collected in line with the methodology recommended by the World Health Organization (WHO) [14].

Data on psychotropic drugs utilization were analyzed from 2006 to 2021, based on the Anatomical Therapeutic Chemical (ATC) Classification System [15]. Psychotropic drugs are coded as follows: NO6B (antidepressants), NO6D (antidementia drugs), NO6B (psychostimulants and nootropics), NO5A (antipsychotics), NO5B (anxiolytics), NO5C (sedatives and hypnotics), and mood stabilizers which are NO3 (antiepileptics) and N02BF (gabapentinoids) [16,17].

Psychotropic drugs used only in the first few years of the investigated period, or those not in use during the COVID-19 pandemic in Serbia, were excluded from the study (these were: fluvoxamine, agomelatine, thioridazine, sertindole, zuclopenthixol, amisulpride, lithium carbonate, potassium clorazepate, buspirone, brotizolam, zaleplon). Medicines that were used only during the COVID-19 pandemic (desvenlafaxine, idebenone, cariprazine, dexmedetomidine) were also excluded from the study.

### Study outcome

The defined daily dose (DDD) per 1,000 people for each drug was the main outcome measure. The defined daily dose it does not represent the actual prescribed dose (PDD), but a standardized technical measure that serves to analyze drug consumption at the population level, i.e., the average daily dose of a drug used for its principal indication in adults, which does not depend on price, strength or package size. Only one DDD is assigned per ATC code and route of administration (oral, parenteral, inhalation, etc.). Drug utilization data presented as DDDs provide a rough estimate (proxy) of the actual drug intake [14,18].

The value of DDD/1,000 people per day is an estimate of the number of defined daily doses consumed in a population of 1,000 people in one day, and is used as an indicator of drug consumption trends. The collected data were correlated with the number of residents who used the drug during the investigation period. Data on the population size were retrieved from the official records of the Statistical Office of the Republic of Serbia (7,498,001 inhabitants in 2006–6,871,547 inhabitants in 2021) [14].

### Ethical statement

To conduct this study, publicly available data were retrieved from the official website of the ALIMS. Given that secondary aggregated data were used in this research, there were no direct participants in this study and the consent of participants has not been necessary. For this reason the study was exempt from ethical review of the Ethics Committee.

### Data analysis

The IBM SPSS Statistical Package version 19.0 was used to analyze the data. A probability level of p < 0.05 was taken as a margin of statistical significance. To analyze the trend in psychotropic drug use over the observed 16-year period the linear regression models were examined. The independent variable in the model was time (2006–2021). The dependent variable in the model was DDD/1,000 residents for each psychotropic drug per year.

To examine a more detailed dynamic in psychotropic drug use, the joinpoint regression analysis was used (version 4.9.1.0). Joinpoint analysis allows for the identification of statistically significant change points (joinpoints) in time series trends [19,20]. Unlike traditional linear models that assume a constant trend, this method segments the series into multiple linear segments and estimates where significant changes in trend direction occur (e.g., a shift from an increase to a decrease). Each identified change point is statistically tested and included in the model only if it significantly improves the model’s fit to the data.

In our model, the independent variable was time (year: 2006–2021), while the dependent variables were annual drug consumption values expressed as DDD per 1,000 inhabitants per day for each observed group or individual psychotropic drug. A constant error variance was assumed, and the software automatically determined the optimal number of joinpoints, allowing a maximum of two change points for a 16-year time series (in line with the tool’s recommendations). The application of this model allowed us to:

- Quantify the average annual change in drug consumption between identified trend segments,
- Detect potential inflection points that coincide with external events such as the onset of the COVID-19 pandemic,
- Compare consumption patterns throughout the observed period in an objective and statistically grounded manner.

## Results

The linear trend analysis of psychotropic drug utilization in the Republic of Serbia from 2006 to 2021 included a total of 54 psychotropic drugs.

**Fig 1:**
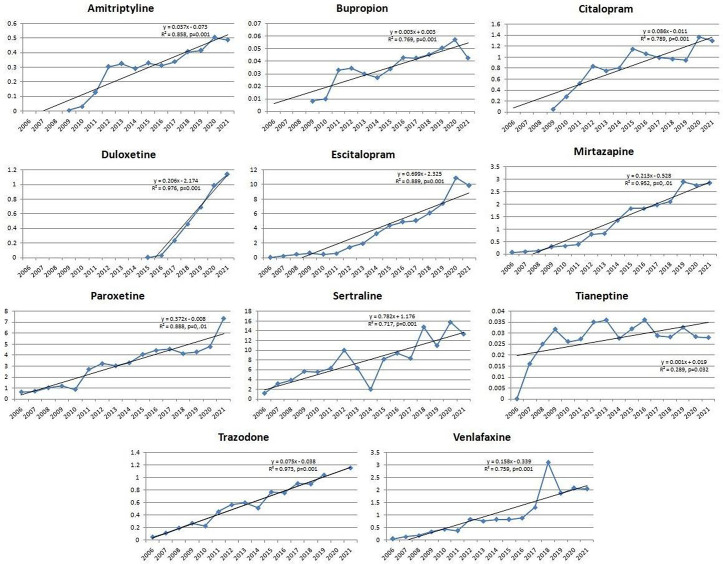
Statistically significant linear increase in use of antidepressants during the observed time period. The increase was evident for amitriptyline, bupropion, citalopram, duloxetine, escitalopram, mirtazapine, paroxetine, sertraline, tianeptine, trazodone, venlafaxine.

**Fig 2:**
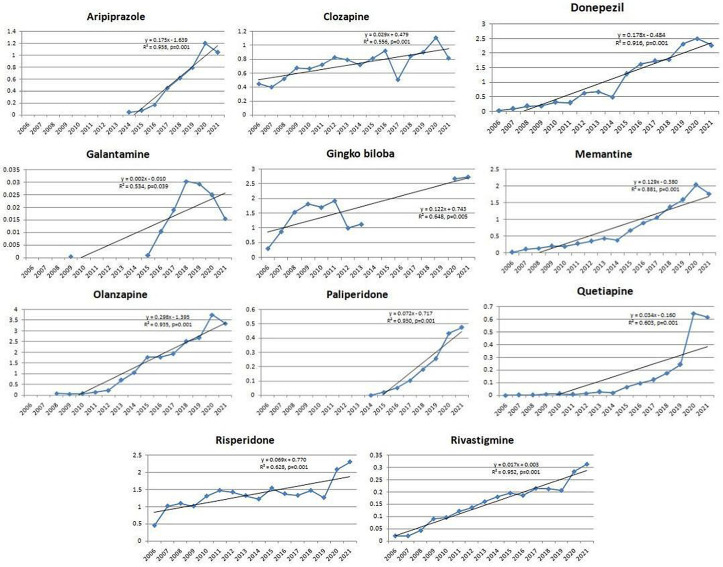
Statistically significant linear increase in use of antidementia medications and antipsychotics during the observed time period. The increase was evident for donepezil, galantamine, ginkgo biloba, memantine, rivastigmine, aripiprazole, clozapine, olanzapine, paliperidone, quetiapine, risperidone.

**Fig 3:**
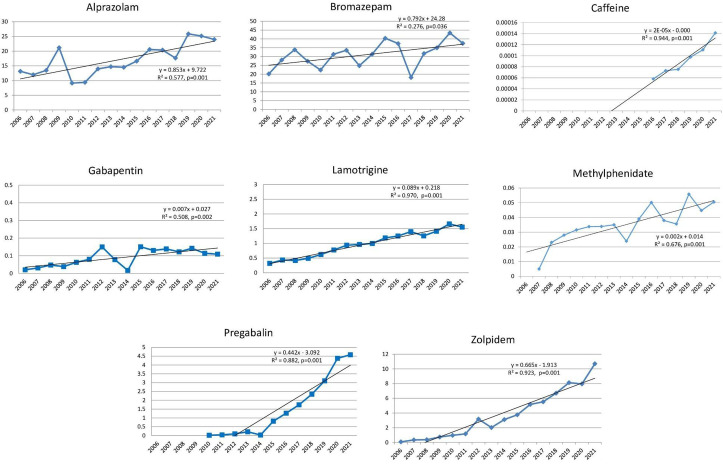
Statistically significant linear increase in use of psychostimulants and nootropic drugs, anxiolytics, sedatives and hypnotics, gabapentinoids during the observed time period. The increase was evident for methylphenidate, caffeine, bromazepam, alprazolam, zolpidem, lamotrigine, gabapentin and pregabalin.

In the observed period (2006–2021) the regression analysis showed a significant linear increase in of:

- Antidepressants – amitriptyline, bupropion, citalopram, duloxetine, escitalopram, mirtazapine, paroxetine, sertraline, tianeptine, trazodone, venlafaxine (Fig 1);
- Antidementia medications – donepezil, galantamine, ginkgo biloba, memantine, rivastigmine (Fig 2);
- Antipsychotics – aripiprazole, clozapine, olanzapine, paliperidone, quetiapine, risperidone (Fig 2);
- Psychostimulants and nootropic drugs – methylphenidate, caffeine (Fig 3);
- Anxiolytics – bromazepam, alprazolam (Fig 3);
- Sedatives and hypnotics – zolpidem (Fig 3);
- Gabapentinoids – lamotrigine, gabapentin and pregabalin (Fig 3).

**Fig 4:**
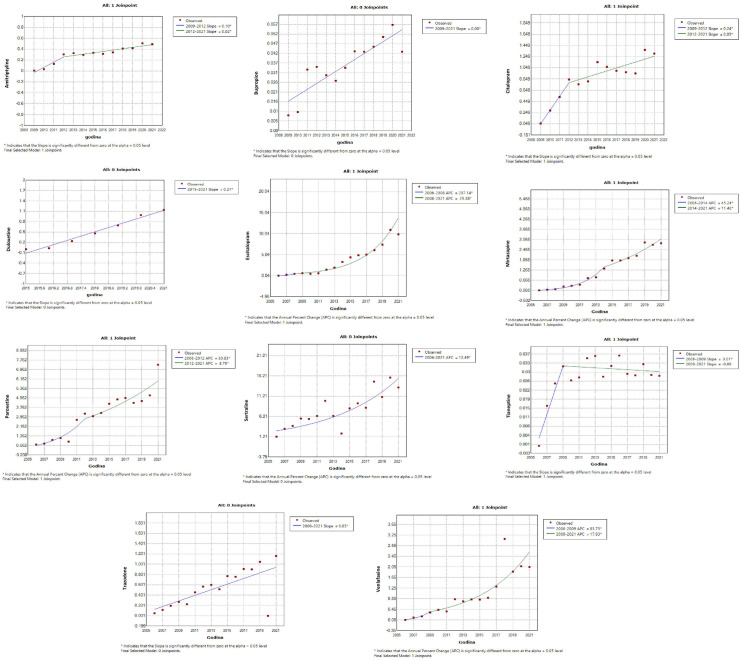
Join-point regression analysis of the statistically significant linear increase in use of antidepressants during the observed time period. The increase before COVID-19 pandemic was particularly evident for tianeptine and escitalopram (from 2008), venlafaxine (from 2009), amitriptyline, citalopram, and paroxetine (from 2012), and mirtazapine (from 2014).

**Fig 5:**
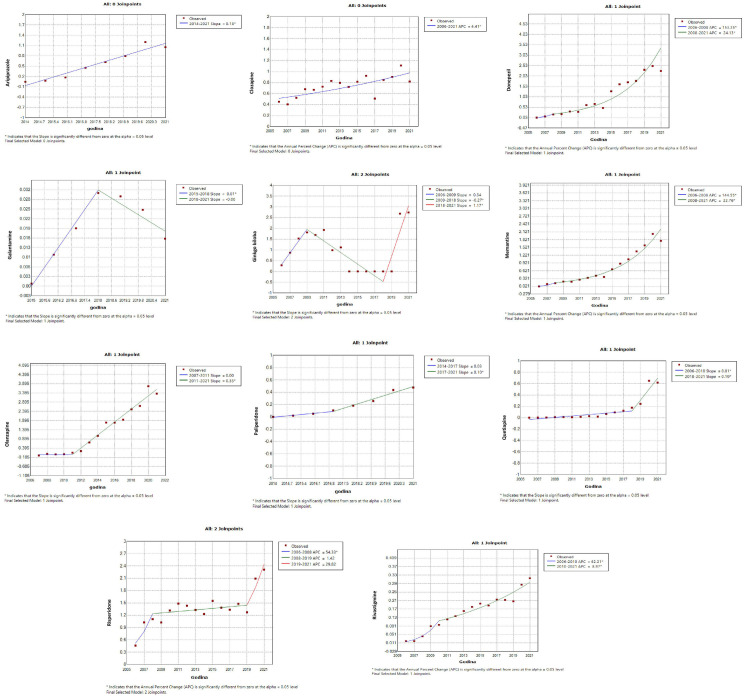
Join-point regression analysis of the statistically significant linear increase in use of antidementia and antipsychotics during the observed time period. Before COVID-19 pandemic among anti-dementia drugs, donepezil showed a steady increase starting from 2008, rivastigmine from 2010, while memantine exhibited a significant increase between 2006 and 2008, followed by a sharp rise thereafter from 2018. Ginkgo biloba showed a significant decline from 2008 to 2018, followed by a sharp increase from 2018 onward. Galantamine usage increased between 2015 and 2018. Regarding antipsychotics, increasing trends were observed for aripiprazole (from 2014), clozapine and risperidone (from 2006), olanzapine (from 2011), paliperidone (from 2017), and quetiapine (from 2018).

**Fig 6:**
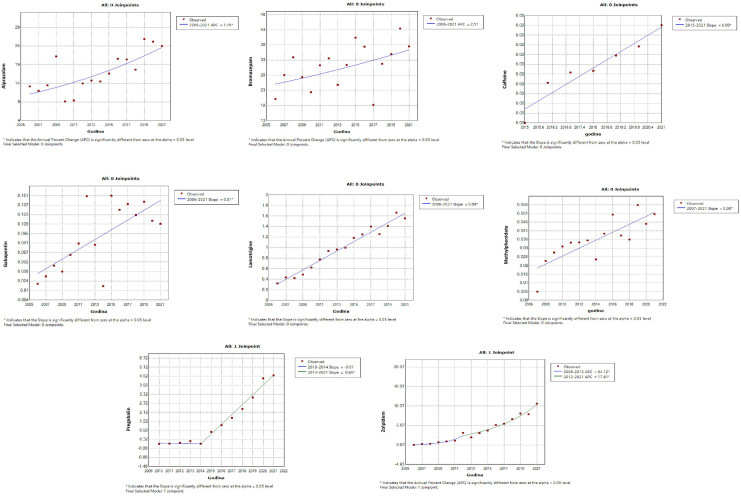
Join-point regression analysis of the statistically significant linear increase in use of anxiolytics, psychostimulants and noot ropic drugs, sedatives and hypnotics, gabapentinoids during the observed time period. Before COVID-19 pandemic mong anxiolytics, alprazolam and bromazepam showed increasing use starting from 2006. Within the group of psychostimulants and nootropic agents, caffeine use increased starting from 2015, and methylphenidate from 2007. The sedative-hypnotic agent zolpidem demonstrated a rising trend beginning in 2012. Among gabapentinoids, gabapentin and lamotrigine showed growth from 2006, while pregabalin usage increased from 2014.

Among drugs that showed a significant linear increase in the trend of utilization, a follow-up joinpoint regression analysis showed that none of the psychotropic drug use changed in 2019 (a cutoff year after which the COVID-19 pandemic was declared). The joinpoint regression analysis registered a significant increase in use of certain psychotropic drugs began much earlier, namely:

- Antidepressants – tianeptine and escitalopram (starting from 2008), venlafaxine (starting from 2009), amitriptyline, citalopram and paroxetine (starting from 2012), mirtazapine (starting from 2014) (Fig 4);
- Antidementia – donepezil (starting from 2008), rivastigmine (starting from 2010), memantine (significant increase 2006–2008, sharp increase starting from 2008), ginkgo biloba (significant decline 2008–2018, sharp increase starting from 2018), galantamine (increase from 2015–2018) (Fig 5);
- Antipsychotics – aripiprazole (starting from 2014), clozapine (starting from 2006), olanzapine (starting from 2011), quetiapine (starting from 2018), paliperidone (starting from 2017), risperideone (starting from 2006) (Fig 5);
- Anxiolytics – alprazolam, bromazepam (satrting from 2006) (Fig 6)
- Psychostimulants and nootropic drugs – caffeine (starting from 2015), methylphenidate (starting from 2007) (Fig 6)
- Sedatives and hypnotics – zolpidem (starting from 2012) (Fig 6);
- Gabapentinoids – gabapentin, lamotrigine (starting from 2006), pregabalin (starting from 2014) (Fig 6).

**Fig 7:**
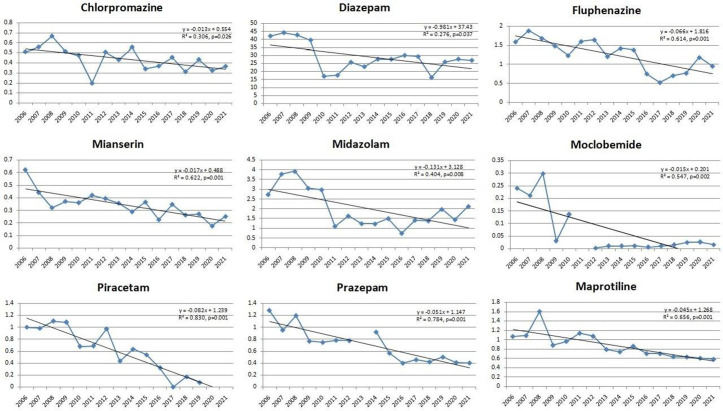
Statistically significant linear decrease in use of antidepressants, antipsychotics, psychostimulants and nootropic drugs, anxiolytics, sedatives and hypnotics during the observed time period. Among antidepressants, decreasing utilization was observed for maprotiline, moclobemide, and mianserin. In the antipsychotic group, chlorpromazine and fluphenazine showed a continuous decline. A similar downward trend was found for the nootropic agent piracetam, the anxiolytics diazepam and prazepam, as well as the sedative-hypnotic midazolam.

On the other hand, in the observed period, the linear regression analysis showed a significant decrease in use of (Fig 7):

- Antidepressants – maprotiline, moclobemide, mianserin;
- Antipsychotics – chlorpromazine and fluphenazine;
- Psychostimulants and nootropic drugs – piracetam;
- Anxiolytics – diazepam and prazepam;
- Sedatives and hypnotics – midazolam.

**Fig 8:**
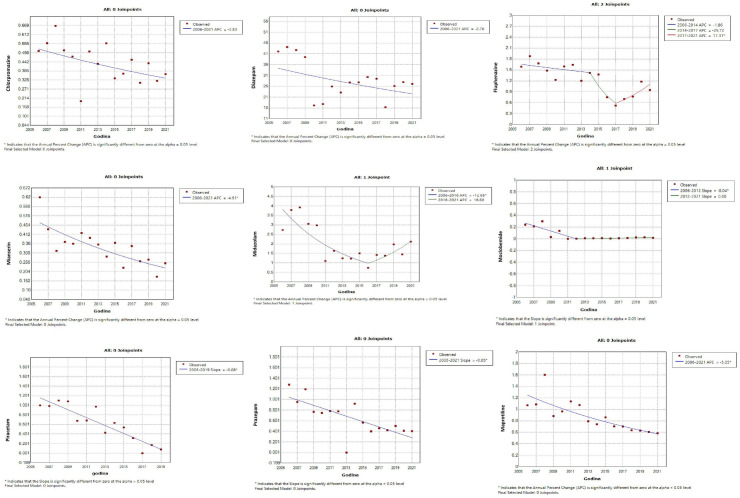
Join-point regression analysis of the statistically significant linear decrease in use of antidepressants, antipsychotics, psychostimulants and nootropic drugs, anxiolytics, sedatives and hypnotics during the observed time period. A significant decline for fluphenazine was observed from 2014 to 2017, followed by a sharp increase starting from 2017.

Of the drugs showing a significant linear decrease in utilization, the subsequent joinpoint analysis showed that only fluphenazine (significant decrease 2014–2017, sharp increase staring from 2017) had changes in use at a specific point in time (Fig 8).

In case of other psychotropic drugs included in this study, the linear regression analysis did not record significant changes in trends (S1A and S1B Fig in S1 File). However, for two psychotropic drugs for which no linear change in trend was registered, such as sulpiride (antipsychotic) and topiramate (antiepileptics), the subsequent jointpoint analysis showed a sudden and statistically significant increase (starting from 2018) and decrease (starting from 2010), respectively (S2A and S2B Fig in S1 File).

## Discussion

This study evaluated the use of 54 psychotropic medications from 2006 to 2021 in the Republic of Serbia. The first two years of the COVID-19 pandemic in Serbia (2020−2021) were also included. Study results showed an increase in consumption of antidepressants, atypical antipsychotics, anxiolytics, sedatives, hypnotics, anti-dementia drugs and gabapentinoids throughout this period. It should be highlighted that the increase in consumption of the analyzed drugs was linear, without any significant changes during the COVID-19pandemic in Serbia.

With regards to antidepressants, this period showed a significant linear increase in consumption of selective serotonin reuptake inhibitors (SSRI), serotonin and norepinephrine reuptake inhibitors (SNRI), bupropion and amitryptiline, which is consistent with the results from other studies [3]. Similar results were reported in Asia and Oceania [21,22]. In Europe, during the period 2000–2010, the prevalence of antidepressants use has doubled, albeit there were considerable differences between regions. In fact, of 27 European countries, a higher prevalence was observed in Scandinavia compared to Eastern Europe, which may be related to socio-cultural and economic factors [23].

In Serbia, the use of SSRIs and SNRIs is favored, which is in line with the current recommendations for treatment of depressive disorders, anxiety disorders, obsessive compulsive disorder (OCD) and post-traumatic stress disorder (PTSD) as well as in treatment of insomnia and pain syndromes [24,25]. Previous studies from Serbia in the period 2013–2015 showed an increase in antidepressants, but not more than in Finland or Norway. At the time, escitalopram was the most commonly prescribed antidepressant in Norway and Finland, whereas sertralin was the most common in Serbia [26]. However, an increase in use of escitalopram was registered in Serbia since 2008, followed by an increase in the use of venlafaxine, amitriptyline, citalopram, paroxetine and mirtazapine. Such a trend might be explained by the change in beliefs and attitudes of people in Serbia about mental illnesses due to the programs to promote mental health as well as broader coverage with psychiatric services [27].

During the first pandemic year a 25% increase in global prevalence of anxiety and depressive disorders was observed [28]. In the US, 4 out of 10 adults exhibited symptoms of anxiety and depression compared to January-June 2019, where there were 1 out of 10 people with comparable symptoms [29]. Evidence from 2021 in Serbia suggests that there was no increase in frequency of diagnosed mental illnesses [30]. A possible explanation may be that long-term exposure to stressors, starting from the 1990s during the civil war in former Yugoslavia, and further economic hardships coupled with chronic political instability triggered the onset of mental illnesses over the early 2000s. It is, therefore, reasonable to assume that those chronic stressors were expectedly more damaging than stress related to COVID-19 pandemic. In terms of antipsychotics, we observed an increase in consumption of atypical antipsychotic drugs and a decrease in consumption of typical antipsychotic drugs. Other studies suggest that, worldwide, over the past 10 years there has been a rise in use of both typical and atypical antipsychotic drugs [3]. Increasing tendency in consumption of olanzapine, quetiapine and paliperidone have been observed across Europe and beyond [31]. A particularly pronounced increase in use of olanzapine, paliperidone and quetiapine coincides with their registration in the Serbian market and their inclusion on the list of medicines covered by health insurance [32–34]. This could explain the change in prescribing practices, introduction of new medicines, primarily atypical antipsychotic drugs in line with the current protocols for treatment of psychotic disorders, while typical antipsychotic drugs use is expectedly reduced [24].

Another reason for increased consumption of atypical antipsychotic drugs may be their favorable adverse events profile compared to the typical ones [35]. Also, potential reasons for increased use of atypical antipsychotic drugs is their use outside the approved indications, such as the treatment of insomnia, and rise in depressive disorder, OCD, anxiety disorders and as antiemetic drugs [36]. Similar to our results, absence of change in psychotropic drug use has been observed in Australia. Given that lockdown in Australia was prolonged, telemedicine was prioritized to facilitate communication between patients and healthcare providers, which may have influenced a steady consumption of psychotropic medications [37].

The results of our study showed an increase in use of benzodiazepines, specifically bromazepam and alprazolom, as well as a hypnotic drug – zolpidem, while diazepam, prazepam and midazolam use declined. In Scandinavia, during the COVID-19 pandemic, an increase in use of anxiolytics and hypnotics was observed compared to the period 2015–2019 [38]. A similar situation was found in Portugal, where older people were more often consumers of anxiolytics, while the consumption of sedatives and hypnotics decreased [39]. Globally, the highest rate of benzodiazepines use was registered in high income countries and the lowest use in middle income countries [40], such as Serbia.

Despite proven effectiveness in treatment of anxiety, agitation and insomnia, the problems with benzodiazepines use are related to adverse events and the possible abuse due to their addictive potential. For example, in older people, they lead to cognitive deficits, increase the risk of falling and mortality [41].

Reduction in use of diazepam and prazepam may be related to side effects of long-acting benzodiazepines. Despite cautions concerning risks associated with long-term benzodiazepine use, a study conducted in the US showed increased consumption of long-term benzodiazepine, especially among older people, [42]. Further, the WHO estimates is that in low and middle income countries 4 out of 5 people do not receive evidence based psychiatric therapy [43]. This is the case in Serbia where benzodiazepine anxiolytics and hypnotics have a long tradition of use outside of the approved psychiatric indications. In fact, it is still possible to obtain psychotropic drugs without prescription, despite the relevant legal regulations.

The COVID-19 pandemic did not affect the trend in use of benzodiazepines in Serbia. This stands in contrast with other studies – for example in Italy since the beginning of the national lockdown an increase in use of benzodiazepines was recorded throughout 2020 [7]. Similar findings were observed in Spain, where increased consumption of sedatives and hypnotics was seen in women, older people and rural areas [44]. In Serbia at this time, a national telephone line for psychological support was created with the aim to help people with their mental problems, particularly anxiety, related to the pandemic [45], which may have helped users and prevented them from using benzodiazepines to soothe.

Of anti-dementia drugs in Serbia, a rise in use was found for donepezil, memantine and rivastigmine, which is in line with the results of the American Population Based Study from 2006 to 2018 [44]. In Italy during 2018–2020 a rise in anti-dementia drugs use was the most prominent among people aged 65–89 years, with no decreasing tendencies in use among other age group except those >90 years [46]. Increase in prescription of supplements, such as ginkgo biloba, since 2018 might be explained by the effects of advertizing and consumers’ economic status, since these supplements have lower market price compared with anti-dementia drugs.

Gabapentinoid pregabalin is indicated as an add-on drug for epilepsy, neuropathic pain and a generalized anxiety disorder [47]. The linear increase in pregabalin consumption in Serbia was observed from 2014 onwards. This may be due to registration of generic products at our market [48] as well as its potential for misuse. A study from Belgium in 2021 and 2022 showed that the highest potential for misuse of pregabalin was among the first generation of migrants, without stable income, suffering from anxiety and depression and who has somatic comorbidities [47]. Therefore, self-medication may be the reason as to why the use of pregabalin was on the rise in Serbia.

### Strengths and limitations

The strength of this study is the fact that the data were used from the official website of ALIMS, which give us the possibility to generalize the results to the entire Serbian population. We were able to analyze a 16-year period, when more than 50 psychotropic drugs were consistently used. In addition, we were able to study multiple psychotropic medications and have a detailed insight into the dynamics of their official prescribing.

This study also has some limitations. It is important to clearly emphasize that this is a secondary analysis of aggregated administrative data, which represents a methodological limitation.The ALIMS, as the national regulatory body, published data on the consumption and turnover of psychotropic medications based on the data collected by pharmaceutical companies without specific indication, duration of treatment, level of health care delivery (primary, secondary, tertiary level; public or private health care sectors). Based on the available data, it is not possible to distinguish whether these drugs were used for approved indications or off-label, or even for self-medication. It is possible that reduced access to health care during COVID-19 pandemic, isolation, reorganization of health care service with all available physicians caring for COVID-19 patients, contributed to a lower access to psychotropic drugs.

## Conclusion

This study showed that the use of antidepressants, atypical antipsychotics, anxiolytics, sedatives, hypnotics, anti-dementia drugs and gabapentinoids increased from 2006 to 2021. Although we cannot differentiate whether there is indeed increase in diagnosed mental health disorders, or the psychiatric services coverage allowed for an increased need for drug-mediated therapy, there seems to be an upward trend in use of psychotropic medications. It is necessary to further follow psychotropic drug dispensing and carefully regulate their use. It is highly recommended to improve mental health promotion, prevention and support interventions in order to decrease drugs consumption in future, especially during public health crisis.

## Supporting information

S1 File **S1A Fig:** Linear regression analysis shows statistically insignificant changes in trends in the consumption of psychotropic drugs in the observed time period. No statistically significant changes in consumption were observed for haloperidol, lorazepam, nitrazepam, oxazepam, topiramate, and ziprasidone over the analyzed period. **S1B Fig:** Linear regression analysis shows statistically insignificant changes in trends in the consumption of psychotropic drugs in the observed time period. No statistically significant changes in consumption were observed for brotizolam, carbamazepine, lithium carbonate, sulpiride, valproate, vinpocetine, zuclopenhixol, fluoxetine, clomipraimne. over the analyzed period. **S2A Fig:** Joinpoint regression analysis shows statistically insignificant changes in trends in the consumption of other psychotropic drugs in the observed time period, except for topiramate (antiepileptics) where it shows a statistically significant decrease. No statistically significant changes in consumption were observed for haloperidol, lorazepam, nitrazepam, oxazepam, and ziprasidone over the analyzed period. **S2B Fig:** Joinpoint regression analysis shows statistically insignificant changes in trends in the consumption of other psychotropic drugs in the observed time period, except for sulpiride (antipsychotic) where it shows a statistically significant increase. No statistically significant changes in consumption were observed for brotizolam, carbamazepine, lithium carbonate, valproate, vinpocetine, zuclopenhixol, fluoxetine, clomipraimne over the analyzed period.
(7Z)
